# *IL17A* and *IL17F* gene polymorphisms in patients with rheumatoid arthritis

**DOI:** 10.1186/s12891-016-1064-1

**Published:** 2016-05-11

**Authors:** Andrzej Pawlik, Daniel Kotrych, Damian Malinowski, Violetta Dziedziejko, Michal Czerewaty, Krzysztof Safranow

**Affiliations:** Department of Physiology, Pomeranian Medical University, Powstancow Wlkp. 72, 70-111 Szczecin, Poland; Departament of Orthopaedics, Traumatology and Orthopaedic Oncology, Pomeranian Medical University, Unii Lubelskiej 1, 71-252 Szczecin, Poland; Department of Pharmacokinetics and Therapeutic Drug Monitoring, Pomeranian Medical University, Powstancow Wlkp. 72, 70-111 Szczecin, Poland; Department of Biochemistry and Medical Chemistry, Pomeranian Medical University, Powstancow Wlkp. 72, 70-111 Szczecin, Poland

**Keywords:** Cytokines, *IL17A*, *IL17F*, Polymorphism, Rheumatoid arthritis

## Abstract

**Background:**

Interleukin-17 plays important role in the pathogenesis of rheumatoid arthritis (RA). The aim of this study was to examine the associations between polymorphisms in the *IL17A* and *IL17F* genes and RA.

**Methods:**

We examined 422 RA patients and 337 subjects as a control group. Single nucleotide polymorphism (SNP) in the *IL17A* (rs2275913) and *IL17F* (rs763780, rs11465553, rs2397084) genes were genotyped using TaqMan genotyping assays from Life Technologies Genomic.

**Results:**

There were no significant differences in distribution of *IL17A* and *IL17F* genotypes and alleles between RA patients and control group. There were no significant associations between *IL17A* and *IL17F* genotypes and age of disease diagnosis rheumatoid factor, erosive disease as well as extra-articular manifestations.

**Conclusions:**

The results of this study suggest, that *IL17A* and *IL17F* gene polymorphism are not the important factors associated with susceptibility and some clinical parameters of RA in a Polish population.

## Background

Rheumatoid arthritis (RA) is a chronic inflammatory disease associated with the destruction of affected joints. Numerous cytokines play the important role in the RA pathogenesis, which initiate and maintenance the inflammatory response in joints.

Interleukin-17A is one member of a cytokine family consisting of six cytokines: IL-17A, IL-17B, IL-17C, IL-17D, IL-17E, and IL-17F. Both IL-17A and IL-17F are secreted by Th17 cells and other immune cells, including innate lymphoid cells [1]. Interleukin-17A is significantly more potent than IL-17F, whereas the IL-17A/IL-17F heterodimer has intermediate activity [2]. Both IL-17A and IL-17F use the IL-17 receptor A (IL-17RA)-IL-17RC heterodimer for their signaling, although IL-17A binds IL-17RA with much higher affinity [3].

IL-17A and IL-17F bind to a receptor complex consisting of two IL-17RA chains and one IL-17RC subunit. IL-17RA is highly expressed on hematopoietic cells, but also on osteoblasts, fibroblasts, endothelial cells, and epithelial cells. In humans, IL-17RA can form a heterodimer with IL-17RC that binds human IL-17A and IL-17F [4]. All IL-17 receptors contain extracellular domains composed of fibronectin type-III domains, and cytoplasmic SEF/IL-17R domains. IL-17 receptor stimulation results in activation of NF-κB and mitogen-activated protein kinases. These signaling properties of IL-17 receptors enable T_H_-17 cells to act as a bridge between innate and adaptive immune cells [5].

The prevalence of IL-17 cells is increased in the circulation of patients with RA; these cells produce higher quantities of IL-17 after stimulation [6]. IL-17 is also present at the sites of inflammatory arthritis and amplifies the inflammation induced by other cytokines and, in particular, TNF-α. In a collagen-induced arthritis (CIA) model, the disease is mainly mediated by IL-17 because IL-17 deficiency, or treatment with IL-17RA antagonist or with IL-17-neutralizing antibody before disease onset, attenuates arthritis with decreased joint damage and reduced serum IL-6 [7]. Previous reports have linked IL-17 to the pathogenesis of RA. The increased expression of IL-17 mRNA and IL-17 protein were detected in joints from RA patients [8, 9]. Moreover, the correlations between serum and synovial fluid levels of IL-17 with various disease activity markers such as erythrocyte sedimentation rate (ESR), C-reactive protein (CRP), and rheumatoid factor (RF) were shown in RA patients. Likewise, the correlation between serum IL-17 levels and DAS28 was observed [10]. In *IL17* gene several polymorphisms have been detected, that may influence the expression of IL-17. The aim of this study was to examine the association between the polymorphisms in *IL17A* and *IL17F* genes and rheumatoid arthritis.

## Methods

### Subjects

We examined 422 patients (340 female, 82 male, mean age 57.5 ± 12.4 years) with rheumatoid arthritis diagnosed according to the criteria of American College of Rheumatology/European League against Rheumatism [11]. Consenting RA patients treated between 2010 and 2013 in the Department of Rheumatology, County Hospital in Szczecin, Poland were enrolled to the study. The patients with other autoimmunological disease and neoplasmatic diseases were excluded from the study. All subjects were Caucasian from the Pomeranian region of Poland. The subjects enrolled in the study underwent routine biochemical blood analysis, and when required, assays for anticardiolipin antibodies, antinuclear antibodies, and immunological complexes. X-rays of the chest, hands, and feet were obtained in all patients and, when required, radiographs of other joints. These were interpreted by two expert radiologists. The evaluation of the subjects included physical examination, with particular focus on the pattern of joint involvement and the occurrence of extra-articular manifestations (such as vasculitis, anemia, sicca syndrome, amyloidosis, organ involvement) and laboratory features, such as rheumatoid factor (RF). The patients were treated with low doses of methotrexate and glucocorticosteroids. The control group was selected randomly from the population of Pomeranian region of Poland and consisted of healthy Caucasian 337 subjects, (261 female, 76 male) without autoimmunological diseases (mean age 60.6 ± 15.4 years). The study was approved by the ethics committee in Pomeranian Medical University, Szczecin, Poland, and written informed consent was obtained from all subjects.

### Genotyping

DNA was extracted from 200 μL of whole blood samples using a GeneMATRIX Quick Blood DNA Purification Kit (EURx, Poland). SNPs within the *IL17A* (rs2275913) and *IL17F* (rs763780, rs11465553, rs2397084) were genotyped using TaqMan genotyping assays from Life Technologies Genomic. Fluorescence data were captured using a 7500 FAST Real-Time PCR System (Applied Biosystems, USA).

### Statistical analysis

Chi-square or Fisher exact tests were used to compare genotype and allele frequencies between the study groups and to analyze associations of clinical characteristics of RA patients with genotypes. Age at onset of RA was compared between genotype groups with Kruskal-Wallis test. Haploview 4.2 software was used for haplotype analysis, D’ and r^2^ calculation. P < 0.05 was considered statistically significant. The power of the study to detect an association of the analyzed SNPs with presence of RA was estimated using the PS program ver. 3.0.43. The study sample size was sufficient to detect with 80 % probability the true effect size of differences in allele frequencies between groups measured as odds ratio (OR) equal to 0.736 or 1.347 for rs2275913, 0.302 or 2.106 for rs763780, 0.435 or 1.822 for rs11465553 and 0.593 or 1.543 for rs2397084.

## Results

### The distribution of *IL17A* and *IL17F* genotypes and alleles

The distributions of *IL17A* and *IL17F* genotypes were in Hardy-Weinberg equilibrium (HWE) and are shown in Table 1. As shown in the Table 1 there were no significant differences in distribution of *IL17A* and *IL17F* genotypes and alleles between RA patients and control group.

**Table 1:** The distribution of *IL17A* and *IL17F* genotypes in RA patients and control group

	RA patients		Control group		*p* ^a^		*p* ^b^	OR (95 % CI)
	*n*	%	*n*	%				
*IL17A* rs2275913 genotype								
GG	173	41.49	118	35.01	0.17	AA + AG vs GG	0.072	0.76 (0.56-1.02)
AG	193	46.28	169	50.15		AA vs AG + GG	0.33	0.80 (0.53-1.22)
AA	51	12.23	50	14.84		AA vs GG	0.13	0.70 (0.44-1.10)
						AG vs GG	0.13	0.78 (0.57-1.06)
						AA vs AG	0.65	0.89 (0.57-1.39)
*IL17A* rs2275913 allele								
G	539	64.63	405	60.09				
A	295	35.37	269	39.91		A vs G	0.077	0.82 (0.67-1.02)
*IL17F* rs763780 genotype								
TT	385	91.23	318	94.08	0.33	CC + CT vs TT	0.17	1.53 (0.87-2.69)
CT	35	8.29	19	5.62		CC vs CT + TT	1.00	1.60 (0.14-17.77)
CC	2	0.47	1	0.30		CC vs TT	1.00	1.65 (0.15-18.30)
						CT vs TT	0.16	1.52 (0.85-2.71)
						CC vs CT	1.00	1.09 (0.09-12.77)
*IL17F* rs763780 allele								
T	805	95.38	655	96.89				
C	39	4.62	21	3.11		C vs T	0.15	1.51 (0.88-2.59)
*IL17F* rs11465553 genotype								
CC	379	89.81	303	89.64	0.96	TT + CT vs CC	1.00	0.98 (0.61-1.57)
CT	43	10.19	35	10.36		TT vs CT + CC		-
TT	0	0.00	0	0.00		TT vs CC		-
						CT vs CC	1.00	0.98 (0.61-1.57)
						TT vs CT		-
*IL17F* rs11465553 allele								
C	801	94.91	641	94.82				
T	43	5.09	35	5.18		T vs C	1.00	0.98 (0.62-1.55)
*IL17F* rs2397084 genotype								
TT	337	79.86	268	79.29	0.13	CC + CT vs TT	0.86	0.97 (0.68-1.38)
CT	83	19.67	63	18.64		CC vs CT + TT	0.086	0.23 (0.05-1.09)
CC	2	0.47	7	2.07		CC vs TT	0.086	0.23 (0.05-1.10)
						CT vs TT	0.85	1.05 (0.73-1.51)
						CC vs CT	0.079	0.22 (0.04-1.08)
*IL17F* rs2397084 allele								
T	757	89.69	599	88.61				
C	87	10.31	77	11.39		C vs T	0.51	0.89 (0.65-1.24)

^a^χ^2^ test

^b^Fisher exact test

*IL17A* rs2275913, HWE: examined group p = 0.83, control group *p* = 0.43

*IL17F* rs763780, HWE: examined group p = 0.22, control group *p* = 0.27

*IL17F* rs11465553, HWE: examined group p = 0.62, control group *p* = 1.00

*IL17F* rs2397084, HWE: examined group p = 0.29, control group *p* = 0.17

### The parameters of clinical course of RA

The associations between studied polymorphisms and clinical parameters of RA were analyzed. There were no significant associations between *IL17A* and *IL17F* genotypes and age of disease diagnosis, rheumatoid factor, erosive disease as well as extra-articular manifestations (Table 2).

**Table 2:** Analysis of *IL17* genotypes in relation to age of disease diagnosis, presence of rheumatoid factor, erosive disease and extra-articular manifestations in RA patients

Genotype	Age of disease diagnosis			Rheumatoid Factor positive		Erosive RA		Extra-articular manifestations	
	Mean [years]	SD	*p* ^a^	(%)	*p* ^b^	(%)	*p* ^b^	(%)	*p* ^b^
*IL17A* rs2275913									
GG	46.6	13.3	0.56	72.02	0.35	80.23	0.64	14.45	0.25
AG	48.1	13.2		78.61		81.87		19.69	
AA	46.6	12.8		74.00		76.00		11.76	
*IL17F* rs763780									
TT	47.3	13.2	0.69	75.60	0.70	80.16	0.52	16.10	0.14
CT	49.0	12.2		74.29		82.86		28.57	
CC	44.5	33.2		50.00		50.00		0.00	
*IL17F* rs11465553									
CC	47.5	13.4	0.63	75.27	1.00	79.84	0.69	18.21	0.084
CT	46.7	12.0		76.19		83.72		6.98	
*IL17F* rs2397084									
TT	47.6	13.1	0.69	75.08	0.04^*^	80.06	0.78	16.62	0.69
CT	46.4	14.0		78.48		80.49		19.28	
CC	50.5	6.4		0.00		100.00		0.00	

^a^Kruskal-Wallis test

^b^χ2 test or Fisher exact test

^*^Fisher exact test *p* = 0.06 for CC vs TT+CT

### Haplotype analysis

Because *IL17A* and *IL17F* genes are located near chromosome 6, we expected that analyzed genetic variants are in linkage disequlibrium making the haplotypes. Our analysis revealed that 3 pairs of polymorphisms are in total linkage disequlibrium (D’ = 1) and 3 pairs in strong linkage disequlibrium (Table 3). In the studied population we have detected 7 haplotype variants (Table 4). As shown in table 4 there were no statistically significant differences in haplotype distribution between RA patients and control group.

**Table 3:** The linkage disequlibrium between loci

Locus 1	Locus 2	D’	*r* ^2^	Distance (bp)
rs2275913	rs763780	0.897	0.02	50706
rs2275913	rs11465553	0.839	0.023	50725
rs2275913	rs2397084	0.587	0.025	50811
rs763780	rs11465553	1	0.002	19
rs763780	rs2397084	1	0.005	105
rs11465553	rs2397084	1	0.007	86

D’ – Lewontin’s D’

**Table 4:** Haplotype frequencies in RA patients and controls

Haplotype	Frequencies		p*
	RA	Control	
GTCT	0.464	0.424	0.12
ATCT	0.336	0.379	0.08
GTCC	0.089	0.097	0.60
GTTT	0.048	0.049	0.93
GCCT	0.045	0.031	0.16
ATCC	0.014	0.017	0.61
ACCT	0.002	0.003	0.81

^*^chi-square test for difference between groups calculated with Haploview 4.2 software

## Discussion

In this study we analyzed the associations between *IL17A* and *IL17F* gene polymorphisms and RA. There were no statistically significant associations *IL17A* and *IL17F* genotypes and the probability of RA development and clinical parameters of disease. Our results seem to be concordant with data of a large genome-wide association study meta-analysis suggesting that IL17 is not one of RA associated loci [12].

The important role of IL-17 in RA pathogenesis and development of inflammatory status has been confirmed by numerous studies. In the early stages of RA IL-17 contributes to increased angiogenesis by stimulating fibroblast like synoviocytes (FLS) to produce vascular endothelial growth factor (VEGF) [13]. Moreover, IL-17 enhanced the secretion of inflammatory mediators such as IL-6, IL-8, prostaglandin E2 (PGE2), and granulocyte colony stimulating factor (G-CSF) from synovial fibroblasts [14]. IL-17 increased also the secretion of cytokines (IL-1β, TNFa, IL-6) by macrophages upon stimulation with recombinant protein [15]. Synergism between IL-17A and TNF has been shown in synovial fibroblasts and chondrocytes from RA patients [16]. Both IL-17A and TNF up-regulate production of vascular endothelial growth factor in rheumatoid synovial fibroblasts. IL-17A promotes joint inflammation, cartilage degradation and bone erosion, which is consistent with data from experimental models of arthritis [17, 18]. In addition to the increased expression of proinflammatory cytokines, IL-17 increased production of matrix metalloproteinases (MMP)-1, −2, −9, and −13. The IL-17 also stimulates the expression of various chemokines which can recruit neutrophils, macrophages and lymphocytes to the synovium, enhancing inflammation with more severe joint damage [19, 20].

In mice model of arthritis IL-17 enhanced synovial inflammation, and joint destruction [21]. Additionally, the Th17 cytokine increased bone erosion during collagen arthritis in murine synovium. IL-17 significantly increased the RANKL/osteoprotegerin (OPG) ratio [22].

The inhibition of IL-17 also significantly reduced bone erosion in a mouse experimental arthritis model by reducing the levels of RANKL, IL-1, and TNF-α [16]. The bone-destructive role of IL-17 is mainly mediated by enhanced RANKL production by osteoblasts, synovial cells, and mesenchymal stem cells. Kikuta et al. demonstrated that Th17 cells could activate mature osteoclasts into a bone-resorbing state [23]. Thus it is likely that Th17 cells in rheumatoid synovium, not only stimulate osteoclast differentiation by M-CSF and RANKL production in osteoclast-supporting cells via IL-17 secretion, but also directly activate osteoclast bone resorption via cell-cell contact as RANKL-producing T cells.

So far *IL17* gene polymorphisms were not widely investigated in RA patients. Nordang et al. examined the association between *IL17A* gene polymorphisms and RA in patients from Norway and New Zealand [24]. A weak association between RA and the promoter SNP rs2275913 was found in the Norwegian population. This association was not replicated in the RA cohort from New Zealand.

Furuya et al. studied the associations between human leukocyte antigen DRB1 (HLA-DRB1) shared epitope, RANK, RANK ligand, osteoprotegerin (OPG), and interleukin 17 (*IL17*) genotypes with age of disease onset and radiographic progression in Japanese patients with early rheumatoid arthritis (RA) [25]. *IL17* gene (rs3804513) polymorphism was associated with radiographic progression at 2 years.

Paradowska-Gorycka et al. investigated the association between RA and (rs763780, rs2397084) polymorphism of *IL17F* gene in Polish RA patients [26]. Overall, the polymorphisms of the *IL17F* gene were not correlated with susceptibility to RA in Polish population. However, the *IL17F* (rs763780) polymorphism was associated with parameters of disease activity, such as number of tender joints, HAQ score or DAS-28-CRP. Moreover, *IL17F* gene (rs2397084) polymorphism correlated with longer disease duration in patients with RA.

In our study *IL17A* and *IL17F* gene polymorphisms were not the factors associated with susceptibility to RA, moreover there were not the statistically significant associations between these polymorphisms and age of disease diagnosis, rheumatoid factor, joint erosions, extra-articular manifestations.

## Conclusions

The results of this study suggest that *IL17A* and *IL17F* gene polymorphism are not the important factors associated with susceptibility and some clinical parameters of RA in a Polish population. Nevertheless this hypothesis requires further investigations.

### Ethics statement

The study was approved by the local ethics committee, written informed consent was obtained from all subjects (Pomeranian Medical University, Szczecin, Poland, statement KB-0012/175/13).

### Consent for publication

Not applicable.

### Availability of data and materials

The raw data for this study is available on request from the corresponding author.

## Abbreviations

CIA: collagen-induced arthritis
CRP: C-reactive protein
ESR: erythrocyte sedimentation rate
FLS: fibroblast like synoviocytes
IL-17: interleukin-17
MMP: matrix metalloproteinase
OPG: osteoprotegerin
PGE2: prostaglandin E2
RA: rheumatoid arthritis
RF: rheumatoid factor
SNP: single nucleotide polymorphism
VEGF: vascular endothelial growth factor

## References

[1] Korn T, Bettelli E, Oukka M (2009). IL-17 and Th17 cells. Annul Rev Immunol..

[2] Bettelli E, Korn T, Kuchroo V (2007). Th17: the third member of the effector T cell Trilogy. Curr Opin Immunol..

[3] Gaffen S (2009). Structure and signaling in the IL-17 receptor superfamily. Nat Rev Immunol..

[4] Tsai HC, Velichko S, Hung LY, Wu R (2013). IL-17A and Th17 cells in lung inflammation: an update on the role of Th17 cell differentiation and IL-17R signaling in host defense against infection. Clin Dev Immunol..

[5] Ely LK, Fischer S, Garcia KC (2009). Structural basis of receptor sharing by interleukin cytokines. Nat. Immunol..

[6] Li N, Wang JC, Liang TH, Zhu MH, Wang JY, Fu XL, Zhou JR, Zheng SG, Chan P, Han J (2013). Pathologic finding of increased expression of interleukin-17 in the synovial tissue of rheumatoid arthritis patients. Int J Clin Exp Pathol..

[7] Nakae S, Nambu A, Sudo K, Iwakura Y (2003). Suppression of immune induction of collagen-induced arthritis in IL-17-deficient mice. J Immunol..

[8] Chabaud M, Durand JM, Buchs N, Fossiez F, Page G, Frappart L, Miossec P (1999). Human interleukin-17: a T cell-derived proinflammatory cytokine produced by the rheumatoid synovium. Arthritis Rheum..

[9] Moran EM, Mullan R, McCormick J, Connolly M, Sullivan O, Fitzgerald O, Bresnihan B, Veale DJ, Fearon U (2009). Human rheumatoid arthritis tissue production of IL-17A drives matrix and cartilage degradation: synergy with tumour necrosis factor-alpha Oncostatin M and response to biologic therapies. Arthritis Res Ther..

[10] Roşu A, Mărgăritescu C, Stepan A, Muşetescu A, Ene M (2012). IL-17 patterns in synovium, serum and synovial fluid from treatment-naïve, early rheumatoid arthritis patients. Rom J Morphol Embryol..

[11] Aletaha D, Neogi T, Silman AJ (2010). Rheumatoid arthritis classification criteria: an American College of Rheumatology/European League against Rheumatism collaborative initiative. Arthritis Rheum..

[12] Okada Y, Wu D, Trynka G (2014). Genetics of rheumatoid arthritis contributes to biology and drug discovery. Nature.

[13] Ryu S, Lee JH, Kim SI (2006). IL-17 increased the production of vascular endothelial growth factor in rheumatoid arthritis synoviocytes. Clin Rheumatol..

[14] Fossiez F, Djossou O, Chomarat P (1996). T cell interleukin-17 induces stromal cells to produce proinflammatory and hematopoietic cytokines. J Exp Med..

[15] Jovanovic DV, Di Battista JA, Martel-Pelletier J, Jolicoeur FC, He Y, Zhang M, Mineau F, Pelletier JP (1998). IL-17 stimulates the production and expression of proinflammatory cytokines, IL-beta and TNF-alpha, by human macrophages. J Immunol..

[16] Goldberg M, Nadiv O, Luknar-Gabor N, Agar G, Beer Y, Katz Y (2009). Synergism between tumor necrosis factor a and interleukin-17 to induce IL-23 p19 expression in fibroblast-like synoviocytes. Mol Immunol..

[17] Bush KA, Farmer KM, Walker JS, Kirkham BW (2002). Reduction of joint inflammation and bone erosion in rat adjuvant arthritis by treatment with interleukin-17 receptor IgG1 Fc fusion protein. Arthritis Rheum..

[18] Koenders MI, Lubberts E, Oppers-Walgreen B, van den Bersselaar L, Helsen MM, Di Padova FE, Boots AM, Gram H, Joosten LA, van den Berg WB (2005). Blocking of interleukin-17 during reactivation of experimental arthritis prevents joint inflammation and bone erosion by decreasing RANKL and interleukin-1. Am J Pathol..

[19] Azizi G, Jadidi-Niaragh F, Mirshafiey A (2013). Th17 cells in immunopathogenesis and treatment of rheumatoid arthritis. Int J Rheum Dis..

[20] Truchetet ME, Mossalayi MD, Boniface K (2013). IL −17 in the rheumatologist’s line of sight. Biomed Res Int..

[21] Lubberts E, Joosten LA, Oppers B, van den Bersselaar L, Coenen-de Roo CJ, Kolls JK, Schwarzenberger P, van de Loo FA, van den Berg WB (2001). IL-1-independent role of IL-17 in synovial inflammation and joint destruction during collagen-induced arthritis. J Immunol..

[22] Lubberts E, van den Bersselaar L, Oppers-Walgreen B, Schwarzenberger P, Coenen-de Roo CJ, Kolls JK, Joosten LA, van den Berg WB (2003). IL-17 promotes bone erosion in murine collagen-induced arthritis through loss of the receptor activator of NF-kappa B ligand/osteoprotegerin balance. J Immunol..

[23] Kikuta J, Wada Y, Kowada T, Wang Z, Sun-Wada GH, Nishiyama I, Mizukami S, Maiya N, Yasuda H, Kumanogoh A, Kikuchi K, Germain RN, Ishii M (2013). Dynamic visualization of RANKL and Th17-mediated osteoclast function. J Clin Invest..

[24] Nordang GB, Viken MK, Hollis-Moffatt JE, Merriman TR, Førre ØT, Helgetveit K, Kvien TK, Lie BA (2009). Association analysis of the interleukin 17A gene in Caucasian rheumatoid arthritis patients from Norway and New Zealand. Rheumatology (Oxford).

[25] Furuya T, Hakoda M, Ichikawa N, Higami K, Nanke Y, Yago T, Kamatani N, Kotake S (2007). Associations between HLA-DRB1, RANK, RANKL, OPG, and IL-17 genotypes and disease severity phenotypes in Japanese patients with early rheumatoid arthritis. Clin Rheumatol..

[26] Paradowska-Gorycka A, Wojtecka-Lukasik E, Trefler J, Wojciechowska B, Lacki JK, Maslinski S (2010). Association between IL-17F gene polymorphisms and susceptibility to and severity of rheumatoid arthritis (RA). Scand J Immunol..

